# Causal Effects of Enhanced Parenting on Resting-State Graph Properties of Adolescents at Risk for Maltreatment

**DOI:** 10.1016/j.bpsgos.2025.100646

**Published:** 2025-10-29

**Authors:** Marta Korom, Mary Dozier, Hung-Wei Bernie Chen, Elisa Macera, Nim Tottenham, Jeffrey M. Spielberg

**Affiliations:** a Department of Psychological and Brain Sciences, University of Delaware, Newark, Delaware; b Department of Psychology, University of Southern California, Los Angeles, California; c Department of Psychology, Columbia University in the City of New York, New York, New York

**Keywords:** Caregiving adversity, Externalizing, Graph theory, Parenting, Properties, Randomized controlled trial, Resting-state network

## Abstract

**Background:**

In this study, we investigated the sustained causal effects of enhanced early caregiving quality on adolescent brain network properties approximately 11 years after families received an attachment-based parenting intervention.

**Methods:**

Participants included 60 adolescents whose parents were referred by Child Protective Services (CPS) because of risk for child maltreatment and 35 adolescents from families without a CPS history (total *N* = 95). CPS-involved families were randomly assigned to either the target intervention (Attachment and Biobehavioral Catch-up [ABC]) (*n* = 31) or a control intervention (Developmental Education for Families [DEF]) (*n* = 29) before the infants turned 2. During adolescence (mean_age_ = 13.4 years, SD = 0.37), participants underwent a 6-minute resting-state functional magnetic resonance imaging scan.

**Results:**

Graph-theoretical analyses were completed with intervention status as the group-level predictor of interest. Adolescents who received the ABC intervention exhibited distinct global and local network properties compared with the DEF group. The ABC group demonstrated lower current-flow global efficiency and more hierarchical structure, indicating intervention-driven modulation of connectome-wide neurodevelopmental outcomes. Node-specific analyses also indicated intervention effects on clustering coefficients and communicability distances in frontal, limbic, and parietal cortices, suggesting nuanced effects of early interventions on local network properties. Exploratory moderation analyses revealed associations between brain network metrics and externalizing symptoms in the DEF group—indicative of neurobiological risk—that were absent in the ABC and low-risk groups.

**Conclusions:**

The results suggest that the ABC intervention causally shapes the development of the resting-state connectome and associated regulatory health, offering insights into the neural pathways through which early enhanced care may get under the skin of at-risk adolescents.

## Plain Language Statement

Early insensitive care, such as abuse and neglect, disrupts the development of the resting-state connectome, making early intervention critical. This study tested whether the Attachment and Biobehavioral Catch-Up (ABC) parenting intervention delivered in infancy leads to lasting causal effects on adolescent brain development. Youth whose parents received the ABC intervention showed more hierarchically organized brain networks and greater network efficiency than those in a control intervention. In the control intervention but not the ABC group, brain network metrics were linked to behavioral problems, suggesting that ABC promotes functional brain organization that protects against behavioral problems.

High-quality, nurturing parental care during infancy supports the healthy development of the connectome (1, 2, 3), and the absence of such care (e.g., neglect, abuse) poses a serious threat to children’s health and brain development (4). Thus, with parents who are at risk of providing insensitive care, it is critical to intervene early in development to prevent the downstream effects of problematic care (5). The current study leverages data from a longitudinal randomized clinical trial (RCT) that was conducted approximately 11 years after the intervention to examine the causal effects of an early evidence-based parenting program on complex network properties in adolescents at risk for maltreatment.

A key level at which to identify the negative impact of adversity on children’s health is the resting-state functional connectome (resting-state functional magnetic resonance imaging [rs-fMRI]), which reflects low-frequency temporal fluctuations of intrinsic neural activity and interactions between different brain regions (6). Caregiving adversity is associated with disruption in resting-state functional communication between the limbic system (e.g., amygdala, hippocampus, insula) and regions that support top-down regulation (e.g., prefrontal cortex) (7), which support optimal social and emotional health (8). Although informative, this research has mostly examined the connectivity between pairs of nodes without considering the larger network, making it limited in scope because disruption in one connection may be compensated for or associated with further disruptions elsewhere in the network (9,10). Examining the organization of the network can elucidate emergent properties of the entire network and specific nodes as part of a local network (11) and inform us about the impact of insensitive care on brain-wide functional organization (12). Graph theory provides tools for assaying diverse emergent network properties by reducing the vast search space of brain networks in meaningful ways (11). For example, segregation captures how a network is clustered into subnetworks, which is necessary for specialized processing to occur. Integration reflects how efficiently information is distributed across the network. Resilience captures how a network can be vulnerable to disruption. Finally, hierarchy reflects the extent to which nodes are organized into hierarchical levels (10). Together, these metrics reflect specific instantiations of broader categories of network function.

### The Development of Network Properties During Adolescence

The normative development of network properties during adolescence has been studied in resting-state functional networks. This work suggests that network segregation and integration increase with age, and the strength of short-range links declines over time (13), whereas long-range connections and cortical-cortical communication exhibit a steady increase throughout development (13,14). These changes that characterize adolescence reflect a functional specialization of distributed networks and an increasing reliance on higher-order cortical networks rather than subcortical or sensory circuits (13,15, 16, 17).

Disruption in the specialization of these networks has been associated with significant regulatory difficulties in youth. For example, recent work in the Adolescent Brain Cognitive Development (ABCD) Study has suggested that reduced modularity in resting-state and task networks may be a neurobiological marker of externalizing behavior (18). Others have also shown that a loss of segregation between the default mode and executive networks emerged as a correlate of both transdiagnostic internalizing and externalizing problems (19). Together, these findings suggest that alterations in resting-state network properties may underlie a broad spectrum of regulatory difficulties in youth.

A few studies have also examined how retrospective reports of early maltreatment and parenting quality are related to resting-state network properties. For example, the increase in resting-state network integration within the salience network during adolescence mediated the association between maltreatment severity and depressive symptoms and problematic substance use behaviors (20). Nonsocial adversities and increase in social adversity severity have predominantly been associated with efficiency within large-scale resting-state networks (20, 21, 22, 23) and increases in local and global clustering (24). Furthermore, higher network resilience was predictive of better psychosocial resilience in youth with higher cumulative adversity risk (25). A recent study has also identified positive parenting as a moderator between childhood history of abuse and resting-state functional connectivity (rs-FC) between and within canonical resting-state networks, such that increased rs-FC both within and between networks was associated with less positive parenting practices (26). These studies suggest that increases in within-network efficiency (20, 21, 22, 23), local and global segregation (24), and lower network resilience (25) may link the experiences and risks of adversity to impaired self-regulation, with positive parenting being a key mediator (26).

### The Effects of Enhanced Care on Quality of Parenting and Children’s Brain Development

The Attachment and Biobehavioral Catch-up (ABC) intervention (27) is one of the most researched early interventions that is designed to enhance the biobehavioral development of children who are at risk for maltreatment. The active ingredient of ABC is the frequent in-the-moment commenting during sessions (1/min) that supports parents in increasing responsive, nurturing, and sensitive care and reducing frightening behavior when interacting with the infant. ABC has been shown to enhance parental sensitivity and children’s attachment security, emotion regulation (27), executive functioning (28), and functional brain development (29,30). To investigate the effects of ABC on neuromaturation, we conducted a follow-up assessment in middle childhood. We observed intervention effects on amygdala-orbitofrontal cortex (OFC) rs-FC 8 years postintervention. Specifically, both the ABC and low-risk comparison groups showed age-typical near-zero connectivity, whereas the control intervention group of participants who received the Developmental Education for Families (DEF) control intervention exhibited a pattern of negative FC that is more commonly seen in older adolescents (6). Moreover, using tasks designed to identify brain responses associated with caregiver relationships and emotional processing, we have shown that the ABC intervention causally increases activation associated with the representations of maternal cues (30) and enhances top-down regulation of responses to fearful/neutral faces compared with the control intervention group (29). This body of work highlights ABC’s potential to reduce the impact of insensitive and nonresponsive care by intervening early during sensitive periods of development. However, it remains unclear whether and how these developmental benefits are sustained beyond middle childhood, when the prevalence of regulatory problems rapidly increases. By leveraging data from a longitudinal RCT evaluating the efficacy of ABC during adolescence, we begin to address these critical questions.

### The Current Study

In this study, we examined how early parenting interventions following maltreatment risk influence the adolescent resting-state connectome. We collected rs-fMRI data from 13-year-olds whose parents had been randomly assigned to receive the ABC (target) or DEF (control) intervention in infancy following Child Protective Services (CPS) involvement. A non-CPS-involved comparison group was also recruited. Using graph-theoretical analyses, we examined global and local resting-state network properties across the 3 groups (see Table 1 for graph property formulas, definitions, and hypotheses). To contextualize our findings, we conducted exploratory analyses examining the association between network properties and externalizing symptoms followed by an examination of the moderating role of intervention groups on brain-behavior associations.

**Table 1:** Examined Network Properties and Their Formulas, Interpretations, and Hypotheses

Metric	Level	Property	Formula	Interpretation	Hypotheses
Segregation	Local	Clustering coefficient	$C_{i}=\frac{1}{n}\sum_{i\in N}\frac{2t_{i}}{k_{i}\left(k_{i}-1\right)}$	Clustering coefficient is the extent to which the neighbors of a node are also connected to each other, thus forming a more interconnected network around that node. Given that the presence of multiple densely interconnected subnetworks within a larger network is needed for the computation of different types of information simultaneously, higher clustering indicates that the node is more likely to be part of such a subnetwork.	In youth with trauma exposure, Suo *et al.* (24) found a positive association between clustering in the left superior frontal gyrus and trauma symptoms. Thus, we hypothesized that adolescents in the DEF group would exhibit higher clustering coefficients in this region compared with the ABC group.
	Global	Transitivity	$T=\frac{\sum_{i\in N}2t_{i}}{{\sum_{i\in N}k}_{i}\left(k_{i}-1\right)}$	Transitivity reflects the proportion of all possible subnetworks (i.e., triads) that are actually present in the network. Higher transitivity suggests that more densely interconnected subnetworks are present. Given that such subnetworks are needed for the computation of multiple types of specialized processing, higher transitivity suggests that a network has a greater capacity to compute multiple types of information simultaneously.	Suo *et al.* (24) studied trauma-exposed pediatric patients and found greater global clustering (i.e., higher transitivity) in those who developed PTSD. Thus, we hypothesized that the less sensitive early care in the DEF group would be evidenced by higher transitivity compared with the ABC group.
Integration	Local	Communicability distance	$\xi_{i}=\sum_{j}G_{ii}+G_{jj}-2G_{ij}$	Communicability distance reflects the extent to which a node 1) conveys information clearly and 2) with as little waste as possible. Clarity is reflected in the number of possible paths between nodes (i.e., with more paths, the noise from each path will be canceled out), and waste is reflected in the extent to which the information emitted by a node is returned to itself instead of the target nodes.	DEF control treatment has been linked to reduced top-down regulation of threat cues (29), which could be mediated by more efficient transmission of threat-related information. Given that lower levels of communicability distance indicate that a node is a less wasteful and more efficient communicator, we expected that nodes key to threat-processing would show lower communicability distance in the DEF group than in the ABC group.
	Global	Current-flow global efficiency	$E=\frac{1}{nm}\sum_{i=1}^{N}\sum_{j=1,j\neq i}^{N}\frac{1}{R_{ij}}$	Current-flow global efficiency reflects the extent to which a network is able to distribute information as efficiently (i.e., more quickly/strongly) as possible. Higher global efficiency indicates that a network is able to integrate the processing that occurs within subnetworks.	Previous empirical work has shown positive associations between maltreatment severity and resting-state global efficiency (20, 21, 22, 23). Thus, we hypothesized that the protective effects of ABC would be evidenced by lower global efficiency in the ABC group compared with the DEF group.
Centrality	Local	Eigenvector centrality	$\epsilon_{i}=\varkappa_{\lambda_{\max},i}$	Eigenvector centrality captures the extent to which a node is connected to higher-influence nodes (i.e., those with high eigenvector centrality). Influence is a relative quantity, and it is possible to compute because all values are obtained simultaneously via singular value decomposition. Higher values suggest that a node has greater influence over the network via its access to other influential nodes.	Although no work has examined eigenvector centrality in youth at risk for maltreatment, we do have evidence that compared with the ABC group, the DEF group is linked to reduced top-down regulation of threat cues (29), and thus we expected that nodes key to threat processing (e.g., amygdala, hippocampus, insula) would have an increased influence on the network in the DEF group, as evidenced by greater eigenvector centrality.
		Communicability betweenness centrality	${cb}_{i}=\frac{1}{{\left(n-1\right)}^{2}-\left(n-1\right)}\sum_{j}\sum_{k}\frac{{\left(e^{Z}\right)}_{jk}-{\left(e^{Z+E\left(i\right)}\right)}_{jk}}{{\left(e^{Z}\right)}_{jk}}$	Communicability betweenness centrality is the extent to which communication between other nodes flows through the node of interest. Nodes with high communicability betweenness act as intermediaries that facilitate the exchange of information between regions.	In trauma-exposed youth, Suo *et al.* (24) found increased betweenness centrality in superior frontal, prefrontal, and temporal cortices and reduced betweenness centrality in parietal regions in those who developed PTSD. Thus, we hypothesized that the less sensitive early care in the DEF group would be evidenced by greater betweenness centrality in superior frontal, prefrontal, and temporal cortices and reduced betweenness centrality in parietal regions in the DEF group compared with the ABC group.
Resilience	Global	Assortativity	$r=\frac{l^{-1}\sum_{\left(i,j\right)\in L}W_{ij}k_{i}k_{j}-{\left[l^{-1}\sum_{\left(i,j\right)\in L}\frac{1}{2}W_{ij}\left(k_{i}+k_{j}\right)\right]}^{2}}{l^{-1}\sum_{\left(i,j\right)\in L}\frac{1}{2}W_{ij}\left({k_{i}}^{2}+{k_{j}}^{2}\right)-{\left[l^{-1}\sum_{\left(i,j\right)\in L}\frac{1}{2}W_{ij}\left(k_{i}+k_{j}\right)\right]}^{2}}$	Assortativity is the extent to which the nodes in network tend to connect with other nodes that have similar strength (i.e., sum of the weights linked to a node). Higher assortativity indicates that highly connected nodes (hubs) tend to be linked to one another, creating redundancy in the network’s functional architecture. This redundancy makes the network resilient because information can still flow efficiently even if a node/hub is disrupted. Lower assortativity suggests that nodes/hubs are more isolated, making the network more vulnerable to targeted damage.	Based on Bezek *et al.*’s (25) work showing that higher assortativity is associated with psychosocial resilience among youth at high cumulative risk for adversities, we hypothesized that ABC would support similar resilience, as evidenced by higher assortativity, relative to DEF.
Hierarchy	Global	Hierarchical structure	β=−log(C∼k)	Hierarchical structure reflects the extent which nodes with higher strength have lower clustering (coefficient). Such networks will be organized into distinct, tightly clustered subgraphs, connected by fewer, selectively connected hub nodes. This layered structure supports hierarchical information flow, while lower hierarchy suggests a more parallel, distributed mode of processing. Importantly, low hierarchy does not mean disorganization but indicates reliance on parallel integration rather than hierarchical influence.	Given the dearth of research in this area examining hierarchical brain structure, we constructed a hypothesis via the lens of the accelerated neuromaturation hypothesis (1). This theory suggests that early adversity, without a sensitive and responsive caregiver to coregulate the child, promotes earlier maturation of brain networks, allowing children to cope in a more adult style at an earlier age. However, this more rapid trajectory is thought to come with a cost—early maturing systems remain less nuanced and adaptive than those with additional time to experience the world in a protected context, allowing for greater refinement based on such interactions. Given evidence that brain modularity increases from childhood to adolescence (41), we expected the DEF group, which lacks that parental buffering, to evidence a more mature-appearing pattern than the ABC group, namely increased levels of hierarchy. It is important to note that our measure only reflects the extent to which the network is structured hierarchically and does not reflect the level of adaptivity and efficiency engendered by a given structure.

Where Y∼X indicates the regression of Y on X, N = set of all nodes, L = set of all links, n = no. of nodes, *m* = no. of links, A = n × n adjacency matrix wherein each entry = 1 if the corresponding row/column nodes are connected and 0 if not, W = n × n weight matrix wherein each entry is the link weight attached for the corresponding row/column node, $t_{i}=\sum_{j,h\in N}{\left(W_{ij}W_{ih}W_{jh}\right)}^{1/2}$, the node strength $k_{i}=\sum_{j\in N}W_{ij}$, the matrix with node strengths along the diagonal $D=diag\left(k_{i}\right)$, the weight-adjusted adjacency matrix $Z=D^{-1/2}AD^{-1/2}$ , the total network strength $l=\sum_{i,j\in N}W_{ij},E\left(i\right)$ = n × n matrix with entries = 0 except in row and column I and −1 and =−1 in row/column I wherever a link is present in *A*, the pseudo-inverse of Laplacian matrix $L^{+}=\left(pinv\left(D-Z\right)\right)$, the resistance distance matrix $R_{ij}=L_{ii}^{+}+L_{jj}^{+}-2L_{ij}^{+}$, the communicability matrix ${G=e}^{Z}$, $\varkappa_{\lambda_{\max}}$ = the eigenvector associated with the largest eigenvalue associated with the singular value decompensation of Z.

ABC, Attachment and Biobehavioral Catch-up; DEF, Developmental Education for Families; PTSD, posttraumatic stress disorder.

## Methods and Materials

### Participants

Families were referred to the ABC intervention by CPS as part of a foster care diversion program due to risk for maltreatment, including homelessness, neglect, drug use, and possible physical or sexual abuse. Eligible families had infants under 2 years old with no known neurodevelopmental disorders (e.g., Rett syndrome, Down syndrome). Consenting families were randomly assigned to either ABC (target) or DEF (control). Randomization was performed using a random-number table in a parallel design with a 50:50 allocation ratio. Randomization was successful, with no significant pre-intervention differences in demographics or stress hormone regulation (31).

A low-risk comparison group was recruited at age 8 through school and community advertisements. “Low risk” refers to children not referred by CPS who are therefore less likely to have experienced neurodevelopmental disruption from early insensitive care at rates above the general population. While some may have faced adversity, their exposure was presumed to be lower than that of the CPS-involved group. The low-risk comparison group served as a community baseline for evaluating the ABC and DEF groups, not to infer equivalency between the low-risk and the CPS-involved groups when no group differences emerge. Rather, the comparison allows us to examine the extent to which sensitive caregiving mitigates risk pathways and to assess which group (ABC or DEF) more closely resembles the community sample.

All CPS-referred participants who completed the intervention were eligible for follow-up. Of 137 adolescents who were assessed at age 13 years, 95 completed an rs-MRI; 42 were excluded due to refusal, braces, motion, or technical issues (see Supplement Section 1). No demographic differences emerged between participants with and without scans. The sample was racially and ethnically diverse, with most participants identifying as Black or biracial (see Table 2).

**Table 2:** Sociodemographic Characteristics of the Participants

Variables	ABC Group, *n* = 31	DEF Group, *n* = 29	Low-Risk Group, *n* = 35	Group Difference	
				Statistic	*p* Value
Sex, Female	13 (41.9%)	14 (48.3%)	15 (42.9%)	χ^2^_2,__*n*__= 95_ = 0.285	.867
Race					
African-American	21 (67.7%)	19 (65.5%)	13 (37.1%)	χ^2^_8,__*n*__= 95_ = 13.63	.092
Asian-American	1 (3.23%)	0 (0%)	0 (0%)		
Biracial	5 (16.1%)	3 (10.3%)	12 (34.3%)		
White-American	1 (3.23%)	3 (10.3%)	6 (17.1%)		
Other	3 (9.68%)	4 (13.8%)	4 (11.4%)		
Hispanic Ethnicity	3 (9.68%)	6 (20.7%)	8 (22.9%)	χ^2^_2,__*n*__= 95_ = 2.166	.339
Parental Education					
No high school	8 (25.8%)	3 (10.3%)	1 (2.86%)	All 3 groups: χ^2^_10,__*n*__= 95_ = 28.945	.001∗
GED	4 (12.9%)	5 (17.2%)	1 (2.86%)		
High school diploma	10 (32.3%)	14 (48.3%)	9 (25.7%)	Post hoc comparisons: ABC vs. DEF: χ^2^_4,__*n*__= 60_ = 4.589	.332
Some college	9 (29%)	6 (20.7%)	14 (40%)		
4-year college	0 (0%)	1 (3.45%)	7 (20%)	ABC vs. low-risk: χ^2^_5,__*n*__= 66_ = 18.21	.003*∗*
Postgraduate	0 (0%)	0 (0%)	3 (8.57%)	DEF vs. low-risk: χ^2^_5,__*n*__= 64_ = 15.023	.01*∗*∗
Age, Years	13.515 (0.425) [13.019–14.362]	13.405 (0.355) [13.025–14.211]	13.319 (0.324) [12.959–14.096]	*F*_2,92_ = 2.391	.097
Income, USD	$44,042.11 ($31,318.97) [$6000–$130,000]	$27,377.44 ($14,044.45) [$794–$50,000]	$63,577.93 ($64,544.98) [$12,000–$280,000]	All 3 groups: *F*_2,63_ = 3.43	.039∗∗
				Post hoc comparisons: ABC vs. DEF: *B* = −16,665, SE = 15,375, *t* = −1.08	.283
				ABC vs. low-risk: *B* = 19,536, SE = 13,797, *t* = 1.42	.162
				DEF vs. low-risk: *B* = 36,200, SE = 14,026, *t* = 2.581	.012∗∗
Framewise Displacement, mm	0.238 (0.207) [0.027–3.604]	0.237 (0.2) [0.028–4.669]	0.267 (0.222) [0.03–5.392]	*F*_2,92_ = 0.045	.956
CBCL Externalizing T Score	53.03 (10.29)	49.29 (11.14)	46.74 (6.85)	All 3 groups: *F*_2,91_ = 3.662	.03∗∗
				Post hoc comparisons: ABC vs. DEF: *B* = −3.747, SE = 2.463, *t* = −1.521	.131
				ABC vs. low-risk: *B* = −6.289, SE = 2.33, *t* = −2.699	.008∗
				DEF vs. low-risk: *B* = 2.543, SE = 2.395, *t* = 1.062	.291

Values are *n* (%) or mean (SD) [range].

∗*p* < .01, ∗∗*p* < .05.

ABC, Attachment and Biobehavioral Catch-up (active treatment); CBCL, Child Behavior Checklist; DEF, Developmental Education for Families (control treatment); GED, General Education Development Test.

### Procedures

The MRI scans were completed at the Center for Biomedical and Brain Imaging at the University of Delaware using a 64-channel head coil in a 3T Sigma MAGNETOM Prisma Scanner (Siemens). Families received financial incentives to complete the scans (adolescent: $15; parent: $100). All visits followed a structured timeline, and there were no group differences in the start time of resting-state data acquisition (see Supplemental Section 2). Diffusion-weighted images were acquired prior to the resting-state scan. During the resting-state scan, participants viewed a fixation cross and were instructed to stay still, keep their eyes open, avoid falling asleep, and let their minds wander. All participants were treated ethically. The study procedures were approved by the University of Delaware Institutional Review Board.

### Interventions

Both interventions were manualized, 10 sessions long, and delivered in the families’ homes by trained coaches before the infants turned 2 years old.

#### Target Intervention

ABC was designed to enhance the biological and behavioral regulation of young children at risk for receiving insensitive care by encouraging parents to 1) nurture the infant when the infant is distressed, 2) follow the infant’s lead by interacting responsively when the infant is not distressed, and 3) reduce threatening behaviors. Parent coaches frequently commented about the quality of the parent-infant interactions, thus encouraging nurturing and sensitive responses to the child’s cues for engagement (27).

#### Control Intervention

DEF was developed to enhance children’s motor, language, and intellectual development (32). Sessions focused on psychoeducation about children’s early developmental milestones and activities that parents can engage in to enhance their children’s intellectual development. Unlike the original program, DEF did not target responsive care.

### Measures

#### Demographics

We assessed age, sex, race, ethnicity, family income, and caregiver education via self-report. Income data were missing for 29 families (*n*_ABC_ = 12, *n*_DEF_ = 11, *n*_low-risk_ = 6); accordingly, the averages in Table 2 reflect only those who provided income information.

#### Externalizing Symptoms

The Child Behavior Checklist (CBCL) externalizing problems subscale assesses parent-reported behavioral symptoms related to aggression and rule-breaking in children and adolescents age 6 to 18 years (33). This subscale is composed of 35 items. In the current study, the CBCL showed excellent internal consistency (Cronbach’s α = 0.942). T scores were used in all analyses. One participant did not have CBCL data (see Table 2).

#### Network Properties

The list of examined global network properties, their definition, and interpretation is available in Table 1.

### Resting-State Data Processing

#### MRI Data Preprocessing

See Supplement Section 3 for MRI acquisition parameters. Using the FMRIB Software Library (FSL version 6.0.4) (34), the initial preprocessing steps were motion correction, spatial smoothing (5 mm full width at half maximum), and FieldMap correction using topup (FieldMap parameters: echoplanar imaging, 2 × 2 × 2 mm, echo spacing = 0.59 ms; TE = 40 ms). To identify motion artifacts, Independent Component Analysis for Automatic Removal of Motion Artifacts (ICA-AROMA) was applied (35). However, removal of these artifacts was applied to a second set of preprocessed data that were identical except that spatial smoothing was not applied. This was done to improve the separation of signal from adjacent regions of interest (ROIs) because such smoothing would blur signal across ROI boundaries. To ensure that ICA-AROMA successfully removed all visible motion-related variance, we computed differential variance of root-mean-square (DVARS) on the time series after motion components had been removed and flagged any runs in which more than 10% of volumes had a DVARS value that deviated by ≥0.5. Flagged runs were visually inspected for remaining motion-related variance, and if evident, we examined the ICA components that were not identified by ICA-AROMA. Components that appeared motion-related were added to ICA-AROMA’s original list, and component removal was redone, after which the DVARS process described above was redone to determine whether sufficient motion-related variance had been removed. This procedure was performed on 10 participants.

All additional processing steps were completed using the Graph Theory GLM toolbox (GTG) (https://www.nitrc.org/projects/metalab_gtg) version 0.5. Preprocessing steps included second-order polynomial detrending, bandpass filtering (0.01–0.1 Hz), and partialing of nuisance signals, including the mean white matter, ventricular, and global signal. The squared versions of each of these parameters were also included, along with the temporal derivatives of all signals, resulting in a total of 9 nuisance parameters.

#### Computing FC Matrices and Graph Properties

T1-weighted images were processed with FreeSurfer, incorporating T2-weighted images to improve pial surface reconstruction. We then mapped the Human Connectome Project (HCP-MMP1) atlas (36) to each participant’s cortical mantle, converted the atlas surface into 3-dimensional structural space, and merged to create a 370 ROI atlas. Note that the MMPI1 hippocampus ROIs were merged with the segmented hippocampus to create 1 hippocampus ROI per hemisphere. Boundary-based registration was used to transform this atlas from anatomical to functional space.

Following this, the time series for each ROI was extracted by the largest principal component for each ROI, and robust correlations were used to create FC matrices via GTG. Networks were thresholded at 0 to retain only positive links, and the remaining weights were retained in the computation of the graph properties. No sparsity threshold was used because such thresholds are arbitrary and are not needed for weighted networks. Specifically, because of the bias induced by there being no natural weight threshold, past best practice was to compute properties across a range of thresholds and create some representative combination (e.g., area under the curve). However, with weighted matrices, the most representative value is that corresponding to the original nonsparsified matrices. Specifically, small weights will be removed early in the range of thresholds and thus have a correspondingly small influence over property computation, whereas the values obtained by combining across a range of thresholds will be dominated by larger weights because they are retained across more matrices. Thus, in the limit, the influence of each link on the amalgamated property value must approach its influence on the property value obtained from the unthresholded matrix (ignoring the 0 threshold). Finally, 4 global graph properties and 4 nodal properties were computed for each participant’s matrix. Of the 370 ROIs, the nodal properties were calculated for 234 that we hypothesized would be meaningfully relevant for the questions of interest (see Supplemental Section 4 for the list of included ROIs).

### Analytic Plan

Permutation-based (5000 permutations) general linear models were completed using GTG. The main analyses included only the CPS-involved participants, and intervention assignment (ABC vs. DEF) was entered as the main predictor of interest. Because participants were randomly assigned to intervention groups, covariates were excluded from primary analyses because randomization protects against confounding by balancing variables across groups. Given that variance in higher-level properties can be driven by fundamental aspects of the network, we controlled for the global network’s density and total strength for all analyses. For analyses of node-specific properties, we also controlled for that node’s node strength and degree. False discovery rate (FDR) correction was applied across the 234 ROIs included in our network analyses, as well as over the global network metrics tested. FDR correction is widely used and is suitable for large connectivity matrices. Secondary analyses of significant findings examined whether the mean property value for the ABC and DEF groups differed from that of the low-risk comparison group to determine which group was more similar to the low-risk sample of adolescents without a history of CPS involvement.

To contextualize the intervention effects, we conducted exploratory regression analyses in R (version 4.4.2) examining the associations between self-report CBCL externalizing T scores and the local and global graph properties that showed intervention effects. Specifically, with CBCL externalizing T scores as the dependent variable in all models and a graph property as the predictor of interest, with global network density, total strength, and intervention group included as covariates in all analyses and the relevant node degree/strength for node-specific properties. Two regressions were computed for each graph property, with the first testing the main effect of the graph metric and the second testing the interaction between intervention groups (ABC vs. DEF) and the metric. To determine the uniqueness of significant findings, regressions were recomputed with the addition of the other properties (and associated covariates as appropriate) in the model. To aid in the interpretation of interactions, simple slopes were computed (via the *simple_slopes* function in the *reghelper* R package).

All neuroimaging data are made publicly available on the National Institute of Mental Health’s Data Archive. The data analytic code will be made available upon reasonable request from the corresponding author. Information on power analysis is available in Supplemental Section 5.

## Results

### Demographics

See Table 2 for detailed demographics and group statistics. Groups did not differ significantly in age, sex, ethnicity, race, or framewise displacement. Parental education was higher in the low-risk group than in the ABC and DEF groups, and family income differed between the low-risk group and the DEF group but not between the low-risk group and the ABC group.

### Network Properties

Significant intervention effects were found in 2 global and 2 local graph properties. Table 3 summarizes the results and includes the low-risk group. Findings held when controlling for parental education (Supplemental Section 6) and excluding outliers (Supplemental Section 7).

**Table 3:** Intervention Effects on Local and Global Graph Properties

Property Name	Global Graph Properties^a^		Local Graph Properties^b^		
	Current-Flow Global Efficiency	Hierarchical Structure	Clustering Coefficient		Communicability Distance
	NA	NA	Left s6–8	Right Piriform Cortex	Left PFm
Primary Analyses					
ABC (reference group) vs. DEF	ABC < DEF: 5.96∗ [1.82 to 10.09]	ABC > DEF: −0.06∗ [−0.11 to −0.02]	ABC < DEF: 0.05∗∗ [0.02 to 0.08]	ABC < DEF: 0.04∗∗ [0.02 to 0.06]	ABC > DEF: −0.05∗∗ [−0.07 to −0.02]
Follow-Up Analyses					
Low-risk (reference group) vs. ABC	n.s., −1.54 [−5.60 to 2.53]	n.s., 0.01 [−0.04 to 0.06]	n.s., −0.02 [−0.04 to 0.01]	ABC < low-risk: −0.03∗∗∗[−0.05 to −0.01]	n.s., 0.02 [−0.01 to 0.04]
Low-risk (reference group) vs. DEF	Low-risk < DEF: 4.51∗∗∗ [0.47 to 8.55]	Low-risk > DEF: −0.05∗∗∗ [−0.1 to −0.01]	Low-risk < DEF: 0.03∗∗∗ [0.01 to 0.05]	n.s., 0.01 [−0.01 to 0.03]	Low-risk > DEF: −0.03∗∗∗ [−0.05 to −0.00]

Cell entries are unstandardized β-values and their associated 95% CIs. Low-risk indicates the control group without a history of Child Protective Services involvement.

∗*p* < .01, ∗∗*p* < .001, ∗∗∗*p* < .05.

ABC, Attachment and Biobehavioral Catch-up (active treatment); DEF, Developmental Education for Families (control treatment); NA, not applicable; n.s., nonsignificant; PFm, parietal area F, part m, located in the angular gyrus; s6−8, superior portion of the transition area between Brodmann areas 6 and 8 located in the superior frontal gyrus.

a A total of 4 global properties were examined (current-flow global efficiency, hierarchical structures, assortativity, and transitivity), and 2 remained statistically significant after correction for multiple comparisons, resulting in a 50% significance rate.

b A total of 4 local network properties (clustering coefficient, communicability betweenness centrality, eigenvector centrality, and communicability distance) were examined across 234 cortical and subcortical regions. After correction for multiple comparisons, 2 regions remained significant for clustering coefficient and 1 remained significant for communicability distance, corresponding to significance rates of .0085% (2/234) and .0043% (1/234), respectively, and 0% significance percentage for the remaining 2 local network properties that did not yield significant results.

### Global Network Properties

#### Current-Flow Global Efficiency

**Figure 1:**
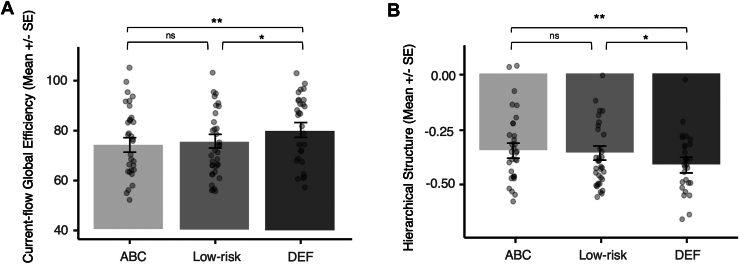
Intervention effects on global graph properties. ∗*p* < .05, ∗∗*p* < .01. Low-risk indicates the control group without a history of Child Protective Services involvement. ABC, Attachment and Biobehavioral Catch-up (active treatment); DEF, Developmental Education for Families (control treatment); ns, nonsignificant.

The ABC group had significantly lower current-flow global efficiency than the DEF group (*b* = 5.96; 95% CI, 1.82–10.09; *R*^2^ = 0.702; *p* = .005). Follow-up analyses indicated that the low-risk group also had lower current-flow global efficiency than the DEF group (*b* = 4.51; 95% CI, 0.47 to 8.55; *R*^2^ = −0.67; *p* = .029), but the low-risk and ABC groups did not differ from each other (*b* = −1.54; 95% CI, −5.60 to 2.53; *R*^2^ = 0.67; *p* = .455) (see Figure 1A).

#### Hierarchical Structure

DEF networks had significantly lower hierarchical structure than ABC networks (*b* = −0.06; 95% CI, −0.11 to −0.02; *R*^2^ = 0.641; *p* = .008). Follow-up analyses indicated that the DEF group was also lower than the low-risk group (*b* = −0.05; 95% CI, −0.10 to −0.01; *R*^2^ = 0.566; *p* = .025), but the low-risk and ABC groups did not differ from each other (*b* = 0.01; 95% CI, −0.04 to 0.06; *R*^2^ = 0.566; *p* = .652) (see Figure 1B).

### Local Network Properties

#### Clustering Coefficient

**Figure 2:**
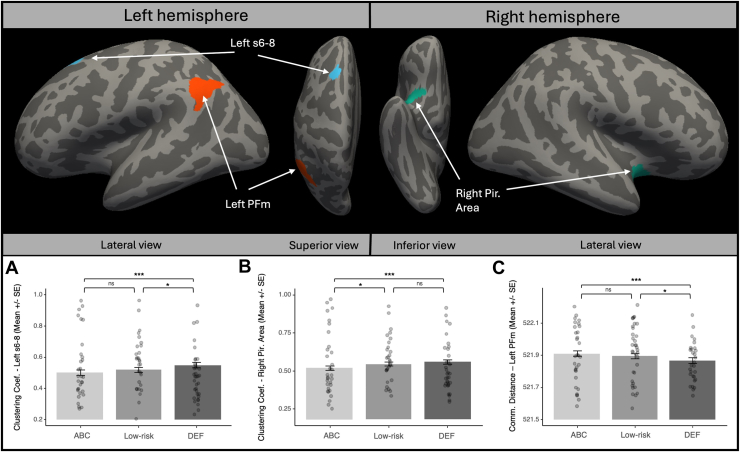
Intervention effects on local graph properties. Cortical areas where intervention effects remained statistically significant after false discovery rate correction for multiple comparisons across all examined nodes are shown on the average inflated brain surface. Corresponding group differences for these areas are displayed in the barplots. ∗*p* < .05, ∗∗∗*p* < .001. Low-risk indicates the control group without a history of Child Protective Services involvement. ABC, Attachment and Biobehavioral Catch-up (active treatment); Coef., coefficient; Comm., communicability; DEF, Developmental Education for Families (control treatment); ns, nonsignificant; PFm, parietal area F, part m, located in angular gyrus; Pir. Area, piriform cortex; s6–8, superior portion of the transition area between Brodmann areas 6 and 8 located in the superior frontal gyrus.

The DEF group had significantly higher left superior frontal gyrus (s6–8) clustering than the ABC group (*b* = 0.05; 95% CI, 0.02 to 0.08; *R*^2^ = 0.938; *p* < .001). Follow-up analyses indicated that the DEF group was also higher than the low-risk group (*b* = 0.03; 95% CI, 0.01 to 0.05; *R*^2^ = 0.937; *p* = .012), but the ABC and low-risk groups did not differ from each other (*b* = −0.02; 95% CI, −0.04 to 0.01; *R*^2^ = 0.937; *p* = .129). The ABC group had significantly lower right piriform clustering than the DEF group (*b* = 0.04; 95% CI, 0.02 to 0.06; *R*^2^ = 0.959; *p* < .001). Follow-up analyses indicated that the ABC group was also lower than the low-risk group (*b* = −0.03; 95% CI, −0.05 to −0.01; *R*^2^ = 0.952; *p* = .011), but the DEF and low-risk groups did not differ from each other (*b* = 0.01; 95% CI, −0.01 to 0.03; *R*^2^ = 0.952; *p* = .177) (see Figure 2A, B).

#### Communicability Distance

The ABC group had significantly higher communicability distance in the parietal area F, part m (PFm) subregion of the left angular gyrus than the DEF group (*b* = −0.05; 95% CI, −0.07 to −0.02; *R*^2^ = 0.926; *p* < .001). Follow-up analyses indicated that the DEF group also had lower values than the low-risk comparison group (*b* = −0.03; 95% CI, −0.05 to −0.00; *R*^2^ = 0.926; *p* = .018), but the ABC and low-risk groups did not differ from each other (*b* = 0.02; 95% CI, −0.01 to 0.04; *R*^2^ = 0.926; *p* = .157) (see Figure 2C).

### Exploratory Analyses

#### Associations With CBCL Externalizing Symptoms

**Figure 3:**
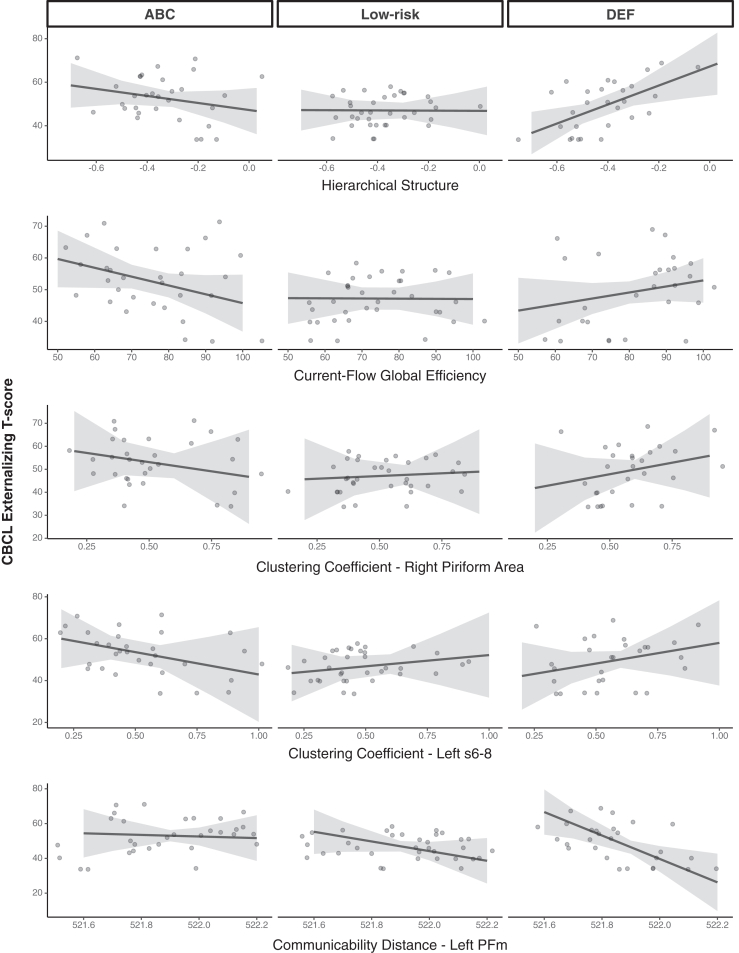
Significant interactions between group and network properties predicting Child Behavior Checklist (CBCL) externalizing subscale T scores. Low-risk indicates the control group without a history of CPS involvement. ABC, Attachment and Biobehavioral Catch-up (active treatment); Coef., coefficient; Comm., communicability; DEF, Developmental Education for Families (control treatment); PFm, parietal area F, part m, located in angular gyrus; Pir. Area, piriform cortex; s6−8, superior portion of the transition area between Brodmann areas 6 and 8 located in the superior frontal gyrus.

No main effects were significant. Intervention group significantly moderated the effect of all properties examined. Current-flow global efficiency, hierarchical structure, and right piriform and left s6-8 clustering showed a more negative association with externalizing symptoms in the ABC group than in the DEF group, with the opposite relationship observed for left PFm communicability distance, which is expected given that this property is keyed in the opposite direction. Figure 3 shows effects across all 3 groups; the low-risk group is included for illustration and had an intermediate profile. See Table 4 for partial correlations, Table 5 for interactions and slopes, and Supplemental Section 8 for the same with the low-risk group included.

**Table 4:** Partial Correlations Between Residualized Network Properties Showing Intervention Effects

		Global Graph Properties		Local Graph Properties		
		Current-Flow Global Efficiency	Hierarchical Structure	Clustering Coefficient - Right Piriform cortex Area	Clustering Coefficient - Left s6–8	Communicability Distance - Left PFm
Global Graph Properties	Current-flow global efficiency	1	–	–	–	–
	Hierarchical structure	−0.32∗	1	–	–	–
Local Graph Properties	Clustering coefficient-right piriform cortex Area	0.49∗∗	−0.26∗	1	–	–
	Clustering coefficient-left s6-8	0.42∗∗	−0.15	0.38∗∗∗	1	–
	Communicability distance-left PFm	−0.52∗∗	0.37∗∗∗	−0.49∗∗	−0.31∗	1

Covariates included global network density and total strength for all analyses. For analyses of node-specific properties, we also controlled for that node’s node strength and degree.

∗*p* < .05, ∗∗*p* < .001, ∗∗∗*p* < .01.

PFm, parietal area F, part m, located in angular gyrus; s6−8, superior portion of the transition area between Brodmann areas 6 and 8 located in superior frontal gyrus.

**Table 5:** Statistical Summary of Intervention Group by Network Property Interactions Predicting CBCL Externalizing T Scores

Property	Effect	Terms	Estimate	SE	*t* Value	*p* Value
Hierarchical Structure	Interaction	Intercept	−7.51	75.06	−0.10	.923
		ABC vs. DEF	58.68	19.74	2.972	<.001∗∗∗
	Simple slopes	ABC	−8.25	17.28	−0.48	.635
		DEF	50.43	19.04	2.65	.01∗
Current-Flow Global Efficiency	Interaction	Intercept	−1.17	73.79	−0.016	.987
		ABC vs. DEF	0.47	0.204	2.279	.027∗
	Simple slopes	ABC	−0.26	0.22	−1.21	.23
		DEF	0.20	0.20	1.03	.31
Clustering Coefficient-Right Piriform Cortex Area	Interaction	Intercept	−26.81	79.05	−0.339	.736
		ABC vs. DEF	35.06	16.90	2.075	.043∗
	Simple slopes	ABC	16.55	39.31	−0.42	.68
		DEF	18.52	38.07	0.49	.63
Clustering Coefficient-Left s6–8	Interaction	Intercept	−54.37	80.28	−0.677	.501
		ABC vs. DEF	42.37	14.48	2.927	.005∗∗
	Simple slopes	ABC	−34.59	29.10	−1.19	.24
		DEF	7.78	28.63	0.27	.79
Communicability Distance-Left PFm	Interaction	Intercept	1069.49	16.549	0.065	.949
		ABC vs. DEF	−64.59	19.55	−3.304	.002∗∗
	Simple slopes	ABC	−2.00	31.61	−0.06	.95
		DEF	−66.59	33.21	−2.00	.05∗

ABC served as the reference group in all models. Covariates included global network density and total strength for all analyses. For analyses of node-specific properties, we also controlled for that node’s node strength and degree.

∗*p* < .05, ∗∗*p* < .01, ∗∗∗*p* < .001.

ABC, Attachment and Biobehavioral Catch-up (active treatment); CBCL, Child Behavior Checklist; DEF, Developmental Education for Families (control treatment); PFm, parietal area F, part m, located in angular gyrus; s6−8, superior portion of the transition area between Brodmann areas 6 and 8 located in superior frontal gyrus.

In our fully adjusted sensitivity analyses, the interaction term remained consistently significant, indicating a robust moderation effect and highlighting the unique influence of each property on network organization and behavioral outcomes.

## Discussion

The current study used data from a longitudinal RCT to examine the effects of enhanced early care on adolescents’ brain network properties approximately 11 years after families received the attachment-based ABC intervention. Our analyses provide preliminary evidence of causal effects on network properties during adolescence, with implications for externalizing problems. Adolescents in the ABC group exhibited less efficient information transmission and a more hierarchical structure than those in the DEF group. Node-specific measures showed lower integration around the piriform cortex and superior frontal gyrus and more efficient left angular gyrus communication in the ABC group than in the DEF group. Importantly, the low-risk comparison group showed an intermediate slope, which was more similar to that of the ABC group, suggesting that enhanced care in children at risk for caregiving adversities mitigates the negative impact of maltreatment on brain network organization during adolescence.

Global network topology assesses overall brain organization by capturing emergent characteristics from interactions among all nodes (11). In our study, the ABC and the low-risk groups showed lower current-flow global efficiency than the DEF group. Global efficiency reflects the ease with which information is integrated and transferred across the network (37). Research suggests that global efficiency increases over time (38) and may be further accelerated by adversity exposure (20,21). Therefore, higher DEF global efficiency may reflect accelerated neurodevelopment following insensitive early care. The ABC intervention may slow this neuromaturation down through enhancements in caregiving quality. Although greater efficiency of information communication across the network may at first appear to be advantageous, leaving the DEF group with the better outcome, there are ample reasons to believe that this is not true. Specifically, less efficient communication across the network may reflect an extended period of synapse pruning and network specialization (1), which in turn allows adolescents more time to learn from their environment and develop more nuanced network organization.

Our analyses also revealed intervention effects on global network hierarchy, with the ABC and low-risk groups having greater hierarchy than the DEF group. Stronger hierarchy supports cognitive growth and information integration (39, 40, 41). Lower hierarchy in the DEF group suggests that early insensitive care shapes the connectome toward distributed organization. Importantly, low hierarchical structure does not indicate a lack of organization but rather a structure wherein information processing and integration may rely more on distributed and parallel processing than a hierarchical cascade of influence (42). Therefore, these results suggest that early insensitive care may fundamentally shape how the connectome is organized, with adverse early experiences leading to increased reliance on a more distributed organization, as indicated by lower levels of hierarchical structure in the DEF group, whereas enhanced care following maltreatment, as seen in the ABC group, may allow for the development of hierarchical network structure.

Local network properties focused on the intervention’s impact on specific regions within the network. Significant effects emerged for clustering coefficient (10) and communicability distance (43). Greater clustering in the DEF group than in the ABC and low-risk group suggests higher embedding of the node within subnetworks, possibly reflecting early maturation that may limit refinement based on environmental input. The superior frontal gyrus involvement in higher-level control may support enhanced behavior adjustment in complex environments under early maturation but may increase risk for regulatory difficulties. The advantage of increased clustering around piriform is less clear given its olfactory function (44). However, its subnetwork, including the amygdala, hippocampus, and OFC (45,46), is crucial for affect-based memory formation and learning (47). Tighter clustering may facilitate better affect-based learning but could limit adaptation and increase externalizing symptom risk due to accelerated maturation during infancy and reduced plasticity to environmental input by adolescence.

Lower angular gyrus communicability distance in the DEF group implies more efficient communication compared to the ABC and low-risk groups, that is, higher-quality information is transmitted from that node with less waste. Given its role in attentional processing (48), this may indicate that the angular gyrus is driving the focus of attention to a greater extent in the DEF than in the ABC or comparison groups, potentially compensating for weaknesses elsewhere in the network or reflecting early maturation.

Consistent with evidence that adversity and parenting quality often serve as moderators of brain-behavior health (6,49, 50, 51, 52, 53), we conducted exploratory moderation analyses to anchor our neuroimaging findings in self-reported externalizing outcomes. Positive associations emerged between most network properties and externalizing symptoms in the DEF group, but not in the ABC or low-risk groups (except for communicability distance, which is keyed in the opposite direction), suggesting that without the benefit of ABC, these network properties, except hierarchical structure, may increase risk for externalizing problems in adolescents who are exposed to maltreatment. The nonsignificant associations in the ABC group suggest that these network properties do not yet function as regulatory pathways. Adolescents in ABC may instead rely on other neurobiological mechanisms or benefit more from social buffering by parents or peers than adolescents in the DEF group.

The findings related to global network hierarchy are particularly interesting. On average, adolescents in the DEF group exhibited lower network hierarchy than those in the ABC group. Within the DEF group, hierarchical structure was positively associated with externalizing symptoms, suggesting that reduced hierarchy plays a protective or compensatory role in high-risk environments. This pattern also offers a different perspective on previous work, which has generally found reduced modularity (18) and loss of segregation between canonical resting-state networks (19) and externalizing symptoms. Given the positive association between hierarchy and externalizing symptoms in maltreated youth without the benefits of ABC, our results raise the possibility that a less hierarchically organized network is a protective rather than a risk factor. In other words, a less hierarchically organized network may facilitate more flexible information processing and behavioral regulation, thereby mitigating externalizing outcomes. Future longitudinal research is encouraged to explore the moderating role of parental quality in brain-behavior associations and whether lower modularity and network hierarchy precede reductions in externalizing symptoms (i.e., are protective) versus being downstream of risk exposure or symptom expression.

Importantly, our exploratory results are consistent with previous work (26) showing that parenting quality is an important moderator of resting-state network connectivity in maltreated youth. Although these are cross-sectional associations, and we are limited in our ability to infer developmental trajectories, the results suggest that the ABC intervention may promote resilience against externalizing problems by buffering against the emergence of neurobiological risk pathways.

Several strengths and limitations of the current study should be noted. Key strengths include the study’s prospective, decade-long design and strong ecologic validity. ABC was delivered to children with experiences of neglect, abuse, and homelessness, enhancing generalizability to populations that are most vulnerable to caregiving-related disruptions. Rigorous data quality procedures (e.g., ICA-AROMA) also improved data retention. Although a larger sample size would be beneficial, this remains one of the largest longitudinal RCT samples in the field (54). The low-risk comparison group was recruited at age 8 rather than in infancy; thus, recruitment timing should be considered when interpreting results involving the low-risk group. Finally, resting-state scan duration was relatively short, although it was comparable to scans commonly used in the literature.

### Conclusions

Despite these limitations, we showed that enhanced care following early risk for maltreatment had sustained effects on adolescents’ local and global resting-state network properties ∼11 years after a brief parenting intervention. Given the randomized design, these effects reflect the benefits of the ABC, which is a manualized 10-session program designed to enhance parental sensitivity. The results suggest that early insensitive care leads to more efficient connectome-wide communication during adolescence. Enhanced sensitive care in the ABC group supports the development of a hierarchical network structure, whereas insensitive care in the DEF group produced connectivity patterns more evenly distributed across the connectome. Overall, the ABC intervention may alter the developmental trajectory of the adolescent connectome, providing a potential neural pathway through which early sensitive care enhances behavioral regulation in youth who are at risk for caregiving adversities.

## Supplementary Material

Supplemental Methods, Results, Tables S1–S3

Key Resources Table
