# Supplementary material for: Causal Effects of Enhanced Parenting on Resting-State Graph Properties of Adolescents at Risk for Maltreatment
Source: Biol Psychiatry Glob Open Sci. 2025 Oct 29;6(2):100646. doi: 10.1016/j.bpsgos.2025.100646 (PMC12818129; doi:10.1016/j.bpsgos.2025.100646)
Supplement: Supplemental Methods, Results, Tables S1–S3 [file mmc1.pdf]

SUPPLEMENTARY INFORMATION

Causal Effects of Enhanced Parenting on Resting-State Graph Properties of Adolescents at Risk for Maltreatment

Korom *et al.*

Supplementary materials

Section 1: Participant Inclusion/Exclusion and MRI Scan Completion

Participants for the present study included 137 adolescents who completed a research assessment at 13 years of age. Out of the 137 participants, 95 successfully completed the resting-state MRI scan. 42 participants with missing data included N=18 who refused to get scanned or asked to be taken out of the scanner before the resting-state scan; N=8 who had braces; N=4 were unable to fit comfortably into the bore due to large body size; N=5 fell asleep; N=5 failed data processing due to high motion (e.g., poor registration); N=2 had technical problems with the scanner, such as head coil failure causing signal dropout.

Section 2: Data acquisition time

Data acquisition start times were compared across the three groups. The overall model was not significant, $F(2,92) = 0.93$, $p = .40$. Compared to the ABC group, neither the low-risk comparison group ($\beta = -0.38$, $SE = 0.41$, $t = -0.93$, $p = .35$) nor the DEF group ($\beta = 0.17$, $SE = 0.42$, $t = 0.39$, $p = .70$) showed significant differences in data acquisition start time. The difference between the low-risk and the DEF groups was also not statistically significant ($\beta = 0.543$, $SE = 0.41$, $t = 1.317$, $p = .191$). These results indicate that data acquisition start times did not differ systematically across the three groups in our sample.

Section 3: MRI Acquisition Parameters

Acquisition parameters were consistent with those used in the Human Connectome Project (HCP)[33] and the Adolescent Brain Cognitive Development Study[34]. fMRI: multi-band EPI (MB-EPI) with an MB factor of 8 (length=5min 21sec, 375 volumes, TR=.829s, spatial resolution=2 mm^3 isotropic, TE=40ms). T1: Volume-navigated multi-echo MPRAGE (VNAV-T1) (.8mm isomorphic, TI=1000ms, TR=2500ms, TEs=1.8ms, 3.6ms, 5.39ms, 7.18ms). T2: Volume-navigated T2-weighted (VNAV-T2) (.8mm isomorphic, TI=1000ms, TR=3200ms, TE=564ms).

Section 4:

Table S1. List of ROIs used in graph theory analyses

ROI ID	Label	Description	ROI ID	Label	Description	ROI ID	Label	Description
10	L_THAL	Thalamus	1109	L_FOP4	Frontal OPercular Area 4	2071	R_8BL	Area 8B Lateral
11	L_CAUD	Caudate	1110	L_MI	Middle Insular Area	2072	R_9p	Area 9 Posterior
12	L_PUTA	Putamen	1111	L_Pir	Piriform Cortex	2073	R_10d	Area 10d
13	L_GP	Pallidum	1112	L_AVI	Anterior Ventral Insular Area	2074	R_8C	Area 8C
17	L_HIPP	Hippocampus	1113	L_AAIC	Anterior Agranular Insula Complex	2075	R_44	Area 44
18	L_AMYG	Amygdala	1114	L_FOP1	Frontal OPercular Area 1	2076	R_45	Area 45
26	L_Nacc	Accumbens	1115	L_FOP3	Frontal OPercular Area 3	2077	R_47l	Area 47 lateral
49	R_THAL	Thalamus	1116	L_FOP2	Frontal OPercular Area 2	2078	R_a47r	Area anterior 47r
50	R_CAUD	Caudate	1117	L_PFt	Area PFt	2079	R_6r	Rostral Area 6
51	R_PUTA	Putamen	1118	L_AIP	Anterior IntraParietal Area	2080	R_IFJa	Area IFJa
52	R_GP	Pallidum	1119	L_EC	Entorhinal Cortex	2081	R_IFJp	Area IFJp
53	R_HIPP	Hippocampus	1120	L_PreS	PreSubiculum	2082	R_IFSp	Area IFSp
54	R_AMYG	Amygdala	1122	L_ProS	ProStriate Area	2083	R_IFSa	Area IFSa
58	R_Nacc	Accumbens	1123	L_PeEc	Perirhinal Ectorhinal Cortex	2084	R_p9_46v	Area posterior 9-46v
1003	L_MST	Medial Superior Temporal Area	1127	L_PHA1	ParaHippocampal Area 1	2085	R_46	Area 46
1011	L_FEF	Frontal Eye Fields	1128	L_PHA3	ParaHippocampal Area 3	2086	R_a9_46v	Area anterior 9-46v
1013	L_55b	Area 55b	1129	L_STSda	Area STSd anterior	2087	R_9_46d	Area 9-46d
1015	L_RSC	RetroSplenial Complex	1130	L_STSdp	Area STSd posterior	2088	R_9a	Area 9 anterior
1018	L_IPS1	IntraParietal Sulcus Area 1	1131	L_STSvp	Area STSv posterior	2089	R_10v	Area 10v
1024	L_MT	Middle Temporal Area	1132	L_TGd	Area TG dorsal	2090	R_a10p	Area anterior 10p
1027	L_SFL	Superior Frontal Language Area	1136	L_TF	Area TF	2091	R_10pp	Polar 10p
1033	L_23d	Area 23d	1138	L_PHT	Area PHT	2092	R_11l	Area 11l
1034	L_v23ab	Area ventral 23 a+b	1139	L_PH	Area PH	2093	R_13l	Area 13l
1035	L_d23ab	Area dorsal 23 a+b	1144	L_PGp	Area PGp	2094	R_OFC	Orbital Frontal Complex
1036	L_31pv	Area 31p ventral	1145	L_IP2	Area IntraParietal 2	2095	R_47s	Area 47s
1039	L_23c	Area 23c	1146	L_IP1	Area IntraParietal 1	2097	R_6a	Area 6 anterior
1041	L_24dd	Dorsal Area 24d	1147	L_IP0	Area IntraParietal 0	2098	R_i6_8	Inferior 6-8 Transitional Area
1042	L_24dv	Ventral Area 24d	1148	L_PFop	Area PF opercular	2099	R_s6_8	Superior 6-8 Transitional Area
1044	L_SCEF	Supplementary and Cingulate Eye Field	1149	L_PF	Area PF Complex	2104	R_52	Area 52
1045	L_6ma	Area 6m anterior	1150	L_PFm	Area PFm Complex	2105	R_RI	RetroInsular Cortex
1055	L_6d	Dorsal area 6	1151	L_PGi	Area PGi	2106	R_PFcm	Area PFcm
1056	L_6mp	Area 6mp (SMA)	1152	L_PGs	Area PGs	2107	R_PoI2	Posterior Insular Area 2
1057	L_6v	Ventral Area 6	1156	L_PHA2	ParaHippocampal Area 2	2109	R_FOP4	Frontal OPercular Area 4
1058	L_p24pr	Area Posterior 24 prime	1162	L_31pd	Area 31pd	2110	R_MI	Middle Insular Area
1059	L_33pr	Area 33 prime	1163	L_31a	Area 31a	2111	R_Pir	Pirform Cortex
1060	L_a24pr	Anterior 24 prime	1165	L_25	Area 25	2112	R_AVI	Anterior Ventral Insular Area
1061	L_p32pr	Area p32 prime	1166	L_s32	Area s32	2113	R_AAIC	Anterior Agranular Insula Complex
1062	L_a24	Area a24	1167	L_pOFC	posterior OFC Complex	2114	R_FOP1	Frontal OPercular Area 1
1063	L_d32	Area dorsal 32	1168	L_PoI1	Area Posterior Insular 1	2115	R_FOP3	Frontal OPercular Area 3

1064	L_8BM	Area 8BM	1169	L_Ig	Insular Granular Complex	2116	R_FOP2	Frontal OPercular Area 2
1065	L_p32	Area p32	1170	L_FOP5	Area Frontal Opercular 5	2117	R_PFt	Area PFt
1066	L_10r	Area 10r	1171	L_p10p	Area posterior 10p	2118	R_AIP	Anterior IntraParietal Area
1067	L_47m	Area 47m	1172	L_p47r	Area posterior 47r	2119	R_EC	Entorhinal Cortex
1068	L_8Av	Area 8Av	1179	L_PI	Para-Insular Area	2120	R_PreS	PreSubiculum
1069	L_8Ad	Area 8Ad	1180	L_a32pr	Area anterior 32 prime	2122	R_ProS	ProStriate Area
1070	L_9m	Area 9 Middle	1181	L_p24	Area posterior 24	2123	R_PeEc	Perirhinal Ectorhinal Cortex
1071	L_8BL	Area 8B Lateral	2003	R_MST	Medial Superior Temporal Area	2127	R_PHA1	ParaHippocampal Area 1
1072	L_9p	Area 9 Posterior	2011	R_FEF	Frontal Eye Fields	2128	R_PHA3	ParaHippocampal Area 3
1073	L_10d	Area 10d	2013	R_55b	Area 55b	2129	R_STSda	Area STSd anterior
1074	L_8C	Area 8C	2015	R_RSC	RetroSplenial Complex	2130	R_STSdp	Area STSd posterior
1075	L_44	Area 44	2018	R_IPS1	IntraParietal Sulcus Area 1	2131	R_STSvp	Area STSv posterior
1076	L_45	Area 45	2024	R_MT	Middle Temporal Area	2132	R_TGd	Area TG dorsal
1077	L_47l	Area 47 lateral	2027	R_SFL	Superior Frontal Language Area	2136	R_TF	Area TF
1078	L_a47r	Area anterior 47r	2033	R_23d	Area 23d	2138	R_PHT	Area PHT
1079	L_6r	Rostral Area 6	2034	R_v23ab	Area ventral 23 a+b	2139	R_PH	Area PH
1080	L_IFJa	Area IFJa	2035	R_d23ab	Area dorsal 23 a+b	2144	R_PGp	Area PGp
1081	L_IFJp	Area IFJp	2036	R_31pv	Area 31p ventral	2145	R_IP2	Area IntraParietal 2
1082	L_IFSp	Area IFSp	2039	R_23c	Area 23c	2146	R_IP1	Area IntraParietal 1
1083	L_IFSa	Area IFSa	2041	R_24dd	Dorsal Area 24d	2147	R_IP0	Area IntraParietal 0
1084	L_p9_46v	Area posterior 9-46v	2042	R_24dv	Ventral Area 24d	2148	R_PFop	Area PF opercular
1085	L_46	Area 46	2044	R_SCEF	Supplementary and Cingulate Eye Field	2149	R_PF	Area PF Complex
1086	L_a9_46v	Area anterior 9-46v	2045	R_6ma	Area 6m anterior	2150	R_PFm	Area PFm Complex
1087	L_9_46d	Area 9-46d	2055	R_6d	Dorsal area 6	2151	R_PGi	Area PGi
1088	L_9a	Area 9 anterior	2056	R_6mp	Area 6mp (SMA)	2152	R_PGs	Area PGs
1089	L_10v	Area 10v	2057	R_6v	Ventral Area 6	2156	R_PHA2	ParaHippocampal Area 2
1090	L_a10p	Area anterior 10p	2058	R_p24pr	Area Posterior 24 prime	2162	R_31pd	Area 31pd
1091	L_10pp	Polar 10p	2059	R_33pr	Area 33 prime	2163	R_31a	Area 31a
1092	L_11l	Area 11l	2060	R_a24pr	Anterior 24 prime	2165	R_25	Area 25
1093	L_13l	Area 13l	2061	R_p32pr	Area p32 prime	2166	R_s32	Area s32
1094	L_OFC	Orbital Frontal Complex	2062	R_a24	Area a24	2167	R_pOFC	posterior OFC Complex
1095	L_47s	Area 47s	2063	R_d32	Area dorsal 32	2168	R_PoI1	Area Posterior Insular 1
1097	L_6a	Area 6 anterior	2064	R_8BM	Area 8BM	2169	R_Ig	Insular Granular Complex
1098	L_i6_8	Inferior 6-8 Transitional Area	2065	R_p32	Area p32	2170	R_FOP5	Area Frontal Opercular 5
1099	L_s6_8	Superior 6-8 Transitional Area	2066	R_10r	Area 10r	2171	R_p10p	Area posterior 10p
1104	L_52	Area 52	2067	R_47m	Area 47m	2172	R_p47r	Area posterior 47r
1105	L_RI	RetroInsular Cortex	2068	R_8Av	Area 8Av	2179	R_PI	Para-Insular Area
1106	L_PFcm	Area PFcm	2069	R_8Ad	Area 8Ad	2180	R_a32pr	Area anterior 32 prime
1107	L_PoI2	Posterior Insular Area 2	2070	R_9m	Area 9 Middle	2181	R_p24	Area posterior 24

Section 5: Power analysis

Power analyses were conducted for the NIH grant application to estimate the statistical power needed to detect differences between experimental and control groups for outcomes most central to the proposed hypotheses. For most analyses, appropriate pilot data were available to allow estimates of group means and standard deviations. Power estimates to detect differences in brain circuitry were based on our findings, and represent large effect sizes (Valadez et al., 2020). Power to detect medium or large effect sizes for the full sample of 200 would be .99. Given that we have experienced minimal attrition from early to middle childhood, we expected minimal attrition into adolescence. However, in a worst-case scenario of very high attrition (20%), power to detect medium and large effects would range from .80 to .99 for a full sample of 160, and .70 to .98 for the reduced sample of 96 adolescents with a history of CPS-involvement.

Since the publishing of Valadez et al., 2020 study, two additional neuroimaging studies have been published using the middle childhood data. In task-based fMRI with the same cohort Valadez and colleagues (2023) found large effect sizes despite the smaller sample size than in the present manuscript. In our middle childhood resting-state functional connectivity analysis, also with a smaller sample size, we found a large effect ($d = 1.17$ with N ABC = 21; N DEF = 17; Korom et al., 2023), confirming strong detection power in an even larger sample–such as in the current study.

Section 6: Socioeconomic Status

To address concerns about SES confounding, we conducted sensitivity analyses. To avoid multicollinearity and inflated standard errors—given that parental education and family income were moderately correlated, $r(69) = .406$, 95% CI [.182, .580], $t = 3.55$, $p < .001$—we included only parental education in these sensitivity analyses. Additionally, more parents provided information about their education level than their income, making parental education the more practical choice for maximizing sample size. Finally, parental education differed significantly across groups—with lower educational attainment in both the ABC and DEF groups compared to the low-risk comparison group—whereas income levels were more similar across groups (ABC vs. low-risk did not differ significantly), further supporting the choice to include parental education as our proxy measure for SES.

In all analyses, ABC served as the reference group. Parental education did not significantly predict any of the network properties where intervention effects were found, and the ABC vs. DEF differences remained statistically significant even after controlling for parental education. Note: in addition to the variables shown in the table, models of local graph properties are adjusted for global network density, total strength, node degree, and node strength, whereas models of global graph properties are adjusted for global network density and total strength.

Table S2.

Network Scope	*Property*	*Variable Name*	*Unstd. β*	*SE*	*t-value*	*p-value*
Local	Clustering Coefficient - Left s6-8	(Intercept)	2.237e-02	3.041e-01	0.074	.942
		Low-risk	2.315e-02	1.365e-02	1.696	.094
		DEF	4.852e-02	1.260e-02	3.851	<.001 ***
		Education	-2.565e-03	4.874e-03	-0.526	.6
Local	Clustering Coefficient - Right Pir	(Intercept)	4.473e-01	2.573e-01	1.739	.086
		Low-risk	2.128e-02	1.159e-02	1.836	.07
		DEF	3.994e-02	1.076e-02	3.711	<.001 ***
		Education	3.573e-03	4.176e-03	0.856	.395
Local	Communicability Distance - Left PFm	(Intercept)	5.235e+02	2.887e-01	1813.170	<.001 ***
		Low-risk	-1.522e-02	1.309e-02	-1.162	.248
		DEF	-4.743e-02	1.193e-02	-3.975	<.001 ***
		Education	-3.029e-03	5.023e-03	-0.603	.548
Global	Hierarchical structure	(Intercept)	-1.667e+00	5.802e-01	-2.874	.005 **
		Low-risk	-4.864e-03	2.697e-02	-0.180	.857
		DEF	-6.228e-02	2.513e-02	-2.479	.015 *
		Education	-4.353e-03	9.736e-03	-0.447	.656
Global	Current-Flow Global Efficiency	(Intercept)	-3.323e+01	4.988e+01	-0.666	.507
		Low-risk	1.156e+00	2.319e+00	0.498	.619
		DEF	6.154e+00	2.161e+00	2.849	.005 **
		Education	4.321e-01	8.370e-01	0.516	.607

Section 7: Outliers

We examined the effect of the outliers on our results across all local and global network properties where intervention effects were found. Removing outliers did not change the results.

Global network properties:

a) *Current-flow global efficiency:* no outliers were identified.

b) *Hierarchical structure:* 2 outliers were identified (1 in the DEF and 1 in the low-risk comparison group).

The results did not change when removing these two participants from the dataset (ABC vs. DEF:

Unstd. β = -.068, SE = .024, t(88) = -2.778, p = .007; ABC vs. BIO: Unstd. β = -.017, SE = .024, t(88) = -0.711, p = .479).

Local network properties:

a) *Clustering Coefficient - L_s6_8: 3* outliers were identified, all in the low-risk comparison group. The results did not change when we removed these 3 participants from the dataset (ABC vs. DEF: Unstd. β = .05, SE = .018, t(85) = 4.204, p = < .001; ABC vs. BIO: Unstd. β = -.011, SE = .012, t(85) = -0.941, p = .349).

b) *Clustering Coefficient - Right Pir:* 1 outlier was identified in the DEF group. The results did not change when we removed this participant from the analysis sample (ABC vs. DEF: Unstd. β = .042, SE = .011, t(87) = 3.982, p = < .001; ABC vs. BIO: Unstd. β = -.027, SE = .01, t(87) = 2.63, p = .01).

c) *Communicability distance - Left PFm*: no outliers were identified.

Section 8:

Table S3. Exploratory analyses, interaction effects including ABC, DEF, and the low-risk comparison group.

Property	Effect	Terms	Estimate	SE	t-value	p-value
Hierarchical Structure		Intercept	-30.18	57.15	-0.528	.599
	Interaction	ABC vs. DEF	59.86	17.56	3.410	<.001 **
		ABC vs. Low-risk	15.66	16.12	0.971	.334
		Low-risk vs. DEF	44.2	18.44	2.397	.019 *
	Simple slopes	ABC	-16.17	13.65	-1.18	.24
		Low-risk	-0.51	13.51	-0.04	.97
		DEF	43.69	15.83	2.76	.01 *
Current-Flow Global		Intercept	-10.87	55.58	-0.195	.846
Efficiency	Interaction	ABC vs. DEF	.469	.178	2.627	.01 *
		ABC vs. Low-risk	.273	.173	1.58	.118
		Low-risk vs. DEF	.196	.178	1.102	.273
	Simple slopes	ABC	-0.28	0.17	-1.67	.10
		Low-risk	-0.01	0.15	-0.04	.97
		DEF	0.19	0.16	1.20	.24
Cl. Coef. - Right Pir. Area		Intercept	-26.81	79.05	-0.339	.736
	Interaction	ABC vs. DEF	36.02	14.72	2.448	.016 *
		ABC vs. Low-risk	20.70	12.61	1.641	.104
		Low-risk vs. DEF	15.32	15.40	.995	.323
	Simple slopes	ABC	-15.99	26.79	-0.60	.55
		Low-risk	4.71	25.56	0.18	.85
		DEF	20.04	26.48	0.76	.45
Cl. Coef.- Left s6-8		Intercept	-47.18	56.82	-.83	.409
	Interaction	ABC vs. DEF	-21.38	12.87	3.195	.002 **
		ABC vs. Low-risk	32.12	12.71	2.528	.013 *
		Low-risk vs. DEF	9.00	14.22	0.633	.528
	Simple slopes	ABC	-21.38	22.65	-0.94	.35
		Low-risk	10.74	20.88	0.51	.61
		DEF	19.75	22.50	0.88	.38
Comm. Dist. - Left PFm		Intercept	2409.91	11510.67	.209	.835
	Interaction	ABC vs. DEF	-23.22	17.06	-3.678	<.001***
		ABC vs. Low-risk	-62.77	12.41	-1.872	.065
		Low-risk vs. DEF	-39.55	16.86	-2.346	.021 *
	Simple slopes	ABC	-4.58	22.00	-0.21	.84
		Low-risk	-27.80	21.06	-1.32	.19
		DEF	-67.35	24.06	-2.80	.01 *

Note. ABC served as the reference group in all models, except for the rows where we report on the 'Low-risk vs. DEF' interaction effects. The low-risk comparison group served as the reference across all analyses comparing the low-risk and DEF groups. Covariates included global network density and total strength for all analyses. For analyses of node-specific properties, we also controlled for that node's strength and degree.
* = p < .05; ** = p < .01*** = p < .001 ABC = Attachment and Biobehavioral Catch-up (active treatment); DEF = Developmental Education for Families (control treatment); Low-risk = control group without a history of caregiving adversities; Cl. Coef. = clustering coefficient; Comm. Dist. = communicability distance; SE = standard error; Left s6-8 = left superior portion of the transition area between Brodmann areas 6 and 8 located in superior frontal gyrus; Right Pir = piriform cortex; Left PFm = left parietal area F, part m, located in angular gyrus.

References

1. Valadez EA, Tottenham N, Tabachnick AR, Dozier M. Early Parenting Intervention Effects on Brain Responses to Maternal Cues Among High-Risk Children. *Am J Psychiatry*. 2020;177(9):818-826. doi:10.1176/appi.ajp.2020.20010011
2. Valadez EA, Tottenham N, Korom M, Tabachnick AR, Pine DS, Dozier M. A Randomized Controlled Trial of a Parenting Intervention During Infancy Alters Amygdala-Prefrontal Circuitry in Middle Childhood. *J Am Acad Child Adolesc Psychiatry*. 2024;63(1):29-38. doi:10.1016/j.jaac.2023.06.015
3. Korom M, Tottenham N, Valadez EA, Dozier M. Associations between cortical thickness and anxious/depressive symptoms differ by the quality of early care. *Dev Psychopathol*. 2023;35(1):73-84. doi:10.1017/S0954579421000845
